# The Molecular Crosstalk between the MET Receptor Tyrosine Kinase and the DNA Damage Response — Biological and Clinical Aspects

**DOI:** 10.3390/cancers6010001

**Published:** 2013-12-19

**Authors:** Michaela Medová, Daniel M. Aebersold, Yitzhak Zimmer

**Affiliations:** 1 Department of Radiation Oncology, Inselspital, Bern University Hospital, and University of Bern, 3010 Bern, Switzerland; E-Mail:daniel.aebersold@insel.ch (D.M.A.); 2 Department of Clinical Research, University of Bern, DKF, MEM-E807, Murtenstrasse 35, 3010 Bern, Switzerland

**Keywords:** MET, DNA damage response, ionizing radiation, radiotherapy, radioresistance

## Abstract

Radiation therapy remains an imperative treatment modality for numerous malignancies. Enduring significant technical achievements both on the levels of treatment planning and radiation delivery have led to improvements in local control of tumor growth and reduction in healthy tissue toxicity. Nevertheless, resistance mechanisms, which presumably also involve activation of DNA damage response signaling pathways that eventually may account for loco-regional relapse and consequent tumor progression, still remain a critical problem. Accumulating data suggest that signaling via growth factor receptor tyrosine kinases, which are aberrantly expressed in many tumors, may interfere with the cytotoxic impact of ionizing radiation via the direct activation of the DNA damage response, leading eventually to so-called tumor radioresistance. The aim of this review is to overview the current known data that support a molecular crosstalk between the hepatocyte growth factor receptor tyrosine kinase MET and the DNA damage response. Apart of extending well established concepts over MET biology beyond its function as a growth factor receptor, these observations directly relate to the role of its aberrant activity in resistance to DNA damaging agents, such as ionizing radiation, which are routinely used in cancer therapy and advocate tumor sensitization towards DNA damaging agents in combination with MET targeting.

## 1. Introduction

The receptor tyrosine kinase (RTK) MET is the cell surface receptor for hepatocyte growth factor (HGF) and is primarily expressed on epithelial cells of many organs during embryogenesis and in adulthood, including the liver, pancreas, prostate, kidney, muscle, and bone marrow [1,2]. Despite being tightly regulated, HGF/MET signaling contributes to oncogenesis and tumor progression in numerous cancers. MET was first identified as an oncogene in 1984 as part of a rare translocation with the nucleoporin *translocated promoter region* gene (*TPR*) giving rise to the TPR-MET chimeric oncoprotein [1]. Since this first observation, a variety of additional oncogenic mechanisms that lead to aberrant MET signaling such as overexpression of HGF and/or MET, *MET* gene amplification and point mutations have been described and extensively characterized in preclinical models [3]. Notably, MET aberrant function does not affect only the tumor cells, but may also exert a crucial impact on the tumor microenvironment, enabling tumor growth and systemic dissemination. In that respect, *in vivo* studies have shown that activation of the HGF/MET signaling promotes cell invasiveness and triggers metastases through direct involvement in regulation of angiogenesis [4].

As to clinical observations, deregulated MET pathway, primarily due to overexpression, has been observed in many human epithelial cancers, including lung, breast, ovary, kidney, colon, thyroid, liver, and gastric carcinomas [5,6,7,8,9,10,11,12]. MET overexpression results from transcriptional activation, hypoxia-induced overexpression [13], or amplification of the *MET* gene [14,15,16].

Importantly, genetic alterations, which generate ligand-independent MET mutants have been found in both hereditary and sporadic papillary renal cell carcinomas and involve mutations in the tyrosine kinase domain of MET [17]. Missense mutations in MET have also been identified in ovarian cancer, childhood hepatoblastoma, metastatic head and neck squamous cell carcinomas, and gastric cancer [18,19,20]. In melanoma and thoracic malignancies, MET mutations clustered predominantly in the SEMA and juxtamembrane domains [21]. In addition to overexpression and point mutations, MET deregulated activation could also occur via aberrant ligand-dependent mechanisms. Particularly, both tumor and mesenchymal cells can be responsible for increased HGF production, leading to paracrine and/or autocrine mechanisms for receptor activation [22]. This mechanism of enhanced MET signaling has been shown to be tumorigenic and metastatic in athymic nude mice [23].

The prognostic role of HGF and/or MET has been extensively examined (reviewed in [24]). MET/HGF overexpression patterns have been reported to correlate with increased tumor growth rate and metastasis and overall poor prognosis. Apart of its role in tumor pathogenesis, MET/HGF deregulated function emerges as an important resistance mechanism to targeted therapies against other oncogene systems such as that of the epidermal growth factor receptor (EGFR) (reviewed in [25]). Moreover, an increasing body of evidence is suggesting that apart of controlling biological consequences that are typically associated with signaling of a growth factor receptor, MET signaling may also be wired with critical pathways of the DNA damage response. These findings are extremely important as they may identify aberrant MET function as an important determinant of resistance of tumor cell response to DNA damaging agents (DDAs) widely used in cancer treatment such as ionizing radiation (IR), the main clinical tool of radiation therapy. In the current manuscript, we aim to review the current data linking MET and tumor cells response to IR.

## 2. Results and Discussion

### 2.1. Radiotherapy

Radiation therapy (RT), whose highly efficient tumoricidal effect is elicited primarily through infliction of DNA damage, is an integral clinical modality that uses high-energy radiation such as X-rays, gamma rays, and charged particles for the treatment of numerous solid tumors [26]. According to the National Cancer Institute, approximately half of all cancer patients receive radiation as a part of their treatment. Ongoing technical developments during recent years in both treatment planning and radiation delivery have led to improvements in local control of tumor growth and reductions in toxicity [27]. However, treatment failure, due to resistance mechanisms, which presumably involve activation of DNA damage response (DDR) signaling pathways that account for loco-regional relapse and consequent tumor progression still remain a critical problem [28]. Understanding the biology underlying these resistance mechanisms is essential for the development of novel combination therapies [29].

Unlike systemic treatments, the impact of RT is local. Although RT affects healthy tissue as well, normal cells can usually repair more effectively DNA damage via the activation of repair as well as checkpoint controls machineries, which are severely compromised in cancer cells [26]. RT is used either as a curative approach, or as a palliative way to reduce cancer symptoms for advanced cancers. RT is also practiced on a prophylactic basis, in order to prevent spreading of tumor cells, following for example surgery [30]. Furthermore, RT is often delivered as pre-operative or adjuvant therapy, also in combination with other anticancer therapies (chemoradiotherapy) [31,32].

RT may be primarily administered either by using a beam delivered by an external irradiation source or internally, by inserting a solid radioactive isotope close to or inside the tumor for limited period of time, a method widely known as brachytherapy. In addition and in particular cases, radioactive isotopes, as radioactive iodine ^131^I, are given systemically for the treatment of particular malignant disorders, such as thyroid cancer. External RT is normally given as a series of short treatments by linear accelerators. Dividing the treatment in 30–35 daily fractions (or as hyperfractionated or accelerated therapy) over 6–7 weeks ensures that less damage is inflicted to normal cells than to cancer cells. The damage to normal cells, affecting predominantly tissues in which cells are dividing more rapidly, is mainly temporary, but is a major reason why radiotherapy does have side effects.

As radiation is a local treatment, side effects are usually confined to the treated area. Early effects such as mucositis and affected skin may be seen already few days or weeks following treatment start and may continue for several weeks after treatment has ended. Other consequences such as radiation-related chronic inflammation and tissue fibrosis may not show up until months, or even years later and may include also secondary cancers. Nevertheless, advanced state of the art delivery technologies, such as three-dimensional conformal radiation therapy, intensity modulated radiation therapy, conformal proton beam radiation therapy, stereotactic radiosurgery or brachytherapy do increase RT safety with considerable better results [29].

Nonetheless and despite these progresses, treatment outcome for numerous malignancies remain grim, with distant metastases and locoregional tumor progression being the main reasons for treatment failure. Areas of intense radiation research over the last decade have included modified fractionation, integration of chemotherapy, high-precision beam delivery and biological targeting.

Whether a tumor can be cured by IR depends to a large extent on the number and radiation sensitivity of clonogenic tumor cells [33] but also on various other factors which contribute to the response to radiation such as repopulation, cell cycle control and naturally, the ability to repair damaged DNA. Additionally, it is well established that hypoxic tumors exhibit considerable compromised IR-induced cytotocity [34]. Better understanding of the molecular basis of radiation resistance will hopefully contribute to the identification of molecules driving these processes and, subsequently, to development of pharmacological compounds that can be used for specific targeting. The proof of this concept has been demonstrated using EGFR inhibitors in head and neck cancer, which represents a role model for modern, biologically optimized radiotherapy [29,35]. However, cancer relapse due to therapeutic resistance and development of distant metastases constitutes a major impediment to effective treatment.

### 2.2. The DNA Damage Response

The DDR machinery comprises of multiple coordinated checkpoint and repair pathways, which detect DNA lesions and signal their presence to numerous effectors participating in the DDR to ensure prompt repair of the damaged DNA. The cellular response to DNA damage is divided into processes that essentially signal into checkpoint regulation and apoptosis on one hand and execution of DNA repair on the other hand. The central components of the signal transduction response are the phosphoinositol-3-kinase-like Ataxia telangiectasia and Ataxia telangiectasia and Rad3-related kinases kinases, ATM and ATR, respectively [36,37,38]. Cells harboring DNA damage that maintain intact cell cycle checkpoints such as TP53 usually proceed to a cell cycle arrest to enable accurate DNA repair. If the DNA damage is excessive, cells may undergo apoptosis or senescence. On contrary, cells with compromised checkpoint signaling (e.g., mutated TP53) may continue to cycle, accumulating potentially more DNA damage with an ultimate increase of genomic instability, which eventually can contribute to a more aggressive malignant phenotype [39,40,41].

ATM is activated in response to DSBs and it relocates through an interaction with the MRE11-RAD50-NBS1 (MRN) complex to sites of DNA breaks [42]. ATM molecules exist as inactive dimers that, when recruited to DSBs, dissociate and autophosphorylate on multiple residues including Ser1981. Subsequently, ATM phosphorylates histone H2AX on Ser139 to generate the phosphorylated form γH2AX, as well as many other substrate proteins including Artemis, MDC1, NBS1, TP53, CHK2 and DNA-PKcs. These proteins further activate cell cycle checkpoints and repair pathways.

ATR is recruited to single-stranded DNA (ssDNA) regions, which arise at stalled replication forks or during the processing of bulky lesions such as UV photoproducts [43]. Stalling of DNA polymerases leads to the generation of ssDNA and subsequent interaction with the single-strand binding replication protein A (RPA). The ssDNA-RPA complex recruits the ATR protein through its regulatory subunit ATRIP and it recruits and activates the RAD17 clamp loader. RAD17 loads the PCNA-related 911 complex onto DNA.

Mediator proteins downstream of ATM/ATR kinases serve both as recruiters of additional substrates and as scaffolds for the assembly of protein complexes, which eventually participate in the repair of lesions. In recent years, MDC1, 53BP1, the MRE11-RAD50-NBS1 (MRN) complex, Claspin, BRIT1/MCPH1, BRCA1, and other proteins were discovered and classified as DDR mediators [44]. In addition to signaling, many proteins involved in DDR, including ATM and H2AX, form nuclear foci, marking thereby the sites of DNA damage.

To maintain genome integrity, the cell relies on complex signaling networks to coordinate cell-cycle checkpoints that, in response to DNA damage, allow for the cell cycle to arrest and DNA repair to proceed, or, on the other hand, activate senescence or cell death pathways. The cascade that results in cell cycle arrest and DNA repair is instigated by the activation of ATM and ATR [45,46,47,48,49]. Checkpoint kinase 1 (CHK1) and checkpoint kinase 2 (CHK2) are serine-threonine kinases downstream of ATR/ATM and play a critical role in initiating cell cycle arrest after DNA damage, allowing time for DNA repair and cell survival. In general, cell cycle arrest is achieved by the inactivation of CDC25 phosphatase family members via the phosphorylation by CHK1/CHK2 reducing thereby cyclin-dependent kinase activity needed for cell cycle progression [50]. 

More specifically, after its activation (predominantly by ATR via phosphorylation of Ser317 and Ser345), CHK1 phosphorylates serine residues on the protein phosphatase CDC25a, which leads to CDC25a ubiquitination and proteolysis. This way, the ability of CDC25a to drive the progression through the S-phase is limited. CHK1 also phosphorylates CDC25c, preventing dephosphorylation and activation of CDK1 what finally results in cell cycle arrest in the G2 phase. CHK2 [51] is believed to be activated mainly by ATM by phosphorylation on Thr68 and its effects on the effector proteins CDC25a, CDC25b/c, and TP53 are similar to those mediated by CHK1.

Although CHK1/CHK2 effects on downstream pathways share some similarities and crosstalk between them has been demonstrated in some studies [52], currently it is assumed, that G1 checkpoint is modulated primarily by the ATM-CHK2-TP53 pathway, as the expression of ATR, CHK1, and CDC25a is limited until the cell passes the restriction point [53]. On the other hand, the importance of CHK1 lies in the regulation of the S, intra-S, and the G2-M phase checkpoints. Since the majority of tumors are deficient in the G1 DNA damage checkpoint pathway due to mutations in *TP53* or the loss of this protein, they rely on S and G2 phase checkpoints for DNA repair and survival. Thus, the modulation of the DNA checkpoint pathways, particularly the inhibition of CHL1, offers the potential to sensitize cancer cells to DNA damage induced by chemotherapeutics and radiotherapy [54]. The potency of this anti-cancer approach is being widely tested by the use of several checkpoint kinase inhibitors, from which three (XL-844, AZD7762, and PF00477736) have already entered clinical trials [55].

DNA repair mechanisms have evolved to respond and remove DNA lesions in order to maintain genetic stability. Defects in DNA repair cause hypersensitivity to DDAs, accumulation of mutations in the genome, and ultimately lead to the development of a broad array of genetic disorders, including cancer. Eucaryotic cells have several mechanisms to restore the structural integrity of DNA [56]: *Direct Damage Reversal* involves several protein activities that recognize very specific modified bases, typically methylated, and repair defects (such as O^6^-alkylguanine, O^4^-alkylthymine) by transferring the modifying group from the DNA to themselves. Three Excision Repair (ER) modes include *nucleotide ER* which fixes bulky lesions such as pyrimidine dimers, *base ER* machinery which repairs damaged bases and SSBs, and *mismatch ER*, a multienzyme system which corrects mismatched nucleotides and small loops occurring during replication. *Homologous Recombination* (HR) faithfully repairs DSBs while more often executed *nonhomologous end-joining* (NHEJ) repairs DSBs in an error-prone way.

Recent studies of the consequences of diminished capacity for DNA repair in humans have linked many human diseases with its decreased functionality of DNA repair [57]. For example, inherited defects in mismatch repair enzymes (e.g., MSH2, 3, 6, MLH1) are linked to colon cancer development. Ataxia telangiectasia, a syndrome in which DSB repair kinase ATM is mutated, is connected with leukemia, lymphoma, radiation sensitivity and genome instability, xeroderma pigmentosum, a disease where NER is affected, displays UV sensitivity and a skin cancer phenotype.

Among the DNA repair machineries, primarily BER, HR, and NHEJ were shown to function in repair of radiation-induced DNA lesions. Specifically, BER is involved in processing the base damage, AP sites, multiply damaged sites, and SSBs, and HR and NHEJ play a role in the repair of DSBs.

### 2.3. Growth Factor RTK Systems Involved in Responses to DNA Damage

#### 2.3.1. EGFR

The most characterized RTK system involved in cellular responses to DNA damage inflicted by DDAs such as IR is that of the EGFR. In the 1990s, studies initiated by the laboratory of Rupert Schmidt-Ullrich were the first to observe that irradiation of cancer cells impacts EGFR signaling [58,59,60]. In that respect, this group reported that exposure of A431 squamous carcinoma cells to IR, using clinically relevant doses, activated the tyrosine kinase enzymatic activity of the EGFR as indicated by the receptor ability to undergo autophosphorylation, similarly, albeit to a lesser extent as by its activation by EGF [58]. Moreover, in the same cells, IR led also to activation of the MAPK pathway, which is tightly associated with cell proliferation. Indeed, the same authors found that a single exposure of A431 cells to irradiation doses that ranged between 0.5 and 5 Gy was sufficient to result in cellular growth and proliferation [58]. In one of the first examples that would suggest a radiosensitization potential by means of EGFR targeting, the study has shown that by using the tyrphostin AG1478, one of the first EGFR kinase inhibitors, it was possible to block IR-dependent EGFR downstream signaling and associated tumor cell proliferation. Mechanistically, Dent *et al*. have shown in both A431 cells and in the breast cancer line MDA-MB-231 that IR-related EGFR activation with consequent cellular proliferation and cytoprotective consequences is modulated via an autocrine loop driven by release of the EGFR ligand, transforming growth factor α (TGFα), with subsequent activation of the MAPK and JNK pathways [61]. Neutralization of TGFα by anti-TGFα antibodies or inhibition of MAPK function by the MEK1/2 inhibitors PD98059 and UO126 resulted in radiosensitization towards IR. As what seems to be the first successful *in vivo* xenograft model, Lammering *et al*. have used MDA-MB-231 cells that overexpress a dominant-negative EGFR mutant, EGFR-CD533, to demonstrate the rationale of blocking of the EGFR for tumor radiosensitization in a pre-clinical setup [62]. Although the few studies described so far suggested a link between IR and EGFR activation (Table 1), establishing therefore a sound biological rationale for EGFR targeting as a radiosensitization modality, they still did not proof a direct link between this RTK system and DDR-associated signaling. The first clue for such a potential crosstalk was provided in a 1998 study by Bandyopadhyay *et al*. who demonstrated a physical interaction between the EGFR and the catalytic subunit of DNA-dependent protein kinase (DNA-PK), a major effector of DNA double strand breaks (DSBs) repair via the non-homologous end join repair (NHEJ) pathway [63]. Moreover, by using the EGFR monoclonal antibody C225 (later cetuximab), these authors managed to detect a significant decrease of 75% in DNA-PK nuclear activity, potentially showing that an interference with EGFR may affect an important DDR activity. In a follow-up study by the Harari group, Huang *et al*. demonstrated the *in vivo* radiosensitizing effects of C225 in models of human squamous cell carcinomas when combined with IR [64]. The authors observed that C225 induced a redistribution of DNA-PK from the nucleus to the cytosol, suggesting therefore one possible mechanism whereby C225 may influence the cellular response to radiation via EGFR targeting. In further support of the involvement of EGFR signaling in response to DNA damage, Yacoub *et al*. reported a MAPK-dependent up-regulation of the DNA repair proteins ERCC1 and XRCC1 in human prostate cancer cell lines and that this induction could be decreased via EGFR targeting by A1478 [65]. Additional mechanistic evidence directly linking EGFR with DDR signaling has been provided in 2005 by Dittmann *et al*. who showed an IR-dependent nuclear translocation of an EGFR–DNA-PK complex in human lung cancer cells A459. Exposure of the cells to C225 interrupted DNA-PK translocation and IR-induced phosphorylation with subsequent attenuation of DNA repair and reduced cellular survival [66,67]. Furthermore, by targeting EGFR with the small molecule erlotinib, Li *et al*. demonstrated impairment of DSBs homologous recombination repair via cytoplasmic sequestration of BRCA1, which plays a critical role in this repair pathway [68]. As to the proof of principle for the potential translational benefit of EGFR inhibition for tumor radiosensitization in context of a clinical setup, Bonner *et al*. provided the first evidence in their seminal *N*. *Engl*. *J. Med.* manuscript of 2006 [69]. In this study, cetuximab combined with RT exhibited significant results in patients with head and neck tumors. The authors reported that the combination of the two modalities significantly improved both locoregional tumor growth control as well as overall survival as compared to results seen with each modality alone. 

Before concluding this chapter in which we covered only partially the data that supports the existence of an EGFR-DDR crosstalk signaling, it might be worthwhile mentioning the significance of EGFR mutations to this crosstalk. In that respect, two manuscripts from the Nirodi group focused on the relevance of EGFR mutations to the response of non-small cell lung cancer (NSCLC) cell lines to IR. NSCLCs harboring mutations in the EGFR TK domain represent a subtype of these tumors that exhibit very high sensitivity to EGFR inhibitors, probably due to the addiction towards this RTK system [70,71,72,73,74,75,76]. In a 2006 published article, Das *et al*. have first reported that that NSCLC lines with EGFR TK mutations such as L858R or the E746-E750 deletion mutant exhibit a dramatic increased sensitivity towards IR as compared with tumors cells that express the WT form of the receptor [77]. In a follow-up 2007 study, the same authors reported that this hypersensitivity results from reduced EGFR radioprotective ability due to a defective interaction between mutated EGFR forms with DNA-PK and subsequent IR-related nuclear translocation [78].

Collectively, these as well as findings from other studies provided solid data suggesting that EGFR is a potential regulator of tumor cells response to DNA damage and support the biological rationale for its targeting along with DDAs.

#### 2.3.2. IGF-1R

Apart of the EGFR, several other RTK systems have been associated with cancer cells response to DNA damage (Table 1) and hence are considered as molecular targets for tumor radiosensitization. The insulin-like growth factor 1 receptor (IGF-1R) is an emerging target in molecular oncology as it is overexpressed in many types of human cancers [79,80,81,82,83]. The first indications that IGF-1R may correlate with tumor response to RT originate from a 1997 manuscript by Turner *et al*. who reported that breast tumors with IGF-1R overexpression correlate with early local relapse within 4 years of lumpectomy and irradiation, suggesting that IGF-1R aberrant expression is associated with radioresistance [84]. This conclusion was further supported by the demonstration that IGF-1R overexpression in murine fibroblasts conferred radioresistance. Radiosensitivity was restored by downregulating IGF-1R expression using antisense (AS) oligonucleotides [84]. In a following study in 2001 by Macaulay *et al*., the authors provided evidence that melanoma cells expressing IGF-1R AS are more sensitive to IR. A clue that IGF-1R downregulation may sensitize tumor cells via interference with function of the DDR has been suggested by the same group with the finding that IGF-1R AS-expressing cells exhibited lower levels of the DDR master upstream kinase ATM [85]. Additional solid mechanistic basis in that respect has been reported by Peretz *et al*. also in 2001 [86] by investigating the IGF-1R expression status in A-T cells, which exhibit extreme sensitivity to IR [87]. The study has shown that A-T cells display low levels of IGF-1R and complementation of these cells with ectopic IGF-1R expression conferred cells with higher resistance towards IR. Further data supporting an IGF-1R-DDR link have been furnished by the Reiss group, who demonstrated that IGF-1R signaling controls intracellular trafficking of the RAD51 recombinase, which is a critical homologous recombination effector [88]. All together, these findings serve as evidence-based data supporting a molecular crosstalk between this RTK system and cellular response to RT and provide a biologic rationale for the use of inhibitors for sensitization of IGF-1R-positive tumors towards RT. Indeed, in a 2007 manuscript, Allen *et al*. have shown radiosensitization of tumors cells, particularly of NSCLC origin to IR using A12, a fully humanized anti-IGF-1R monoclonal antibody. A12 was found to inhibit DSBs repair as manifested by sustained expression of γH2AX [89]. Similar results were recently reported by Riesterer *et al*. that usedhead and neck cancer cells [90].

#### 2.3.3. VEGFR, PDGFR, FGFR

Clues suggesting the potential involvement of additional tyrosine kinase receptors such as those for vascular endothelial growth factors (VEGFRs), platelet-derived growth factors (PDGFRs) and fibroblast growth factors (FGFRs) in response of tumor cells to DNA damage emerge particular from studies that demonstrated tumor cells radiosensitization by using inhibitors for these receptors. In that respect for example, SU6668 is a small molecule inhibitor of the angiogenic RTKs, VEGFR2 (Flk-1/KDR), PDGFR*β* and FGFR1 [91]. By employing xenograft models of fibrosarcoma and pancreatic carcinoma cells lines, Griffin *et al*. have reported that SU6668 administration for 2 days followed by a single dose of 15 Gy of X-irradiation was significantly more effective in suppressing tumor growth than either treatment alone [92]. Analogous data have been reported by Ning *et al*. [93]. In 2003, Abdollahi *et al*. reported that IR-associated tumor growth due to VEGF secretion with consequent pro-angiogenic impact on endothelial cells could be attenuated by both SU6668 and SU5416 [94]. Unlike the multi-kinase inhibition properties of SU6668, SU5416 (semaxanib) is a potent and selective inhibitor of VEGFR2 [95,96]. Using a combination of both SU6668 and SU5416 with relatively low IR doses, Timke *et al*. reported better tumor control in xenograft models of prostate cancer and glioblastoma lines [97]. This has been attributed to the high cytotoxicity of the combined treatment on the endothelial cell compartment as judged from the excessive apoptotic rate measured by caspase-3 activity.

**Table 1:** Observations linking RTK systems to cellular DDR.

DDR-related protein	RTK	Mode of modulation
DNA-PK (dsDNA breaks repair by non-homologous end-joining)	EGFR	physical interaction [63], lower DNA-PK nuclear activity [63] and redistribution of DNA-PK from nucleus to cytosol upon EGFRi [64], EGFR in complex with DNA-PK [67], IR-dependent nuclear translocation of EGFR [67], defective interaction with EGFR mutants [78]
ERCC1 (ssDNA breaks repair by nucleotide excision)	EGFR	MAPK-dependent upregulation [65]
XRCC1 (ssDNA breaks repair by base excision)	EGFR	MAPK-dependent upregulation [65]
BRCA1 (dsDNA breaks repair by homologous recombination)	EGFR	cytoplasmic sequestration upon EGFRi [68]
ATM (dsDNA damage breaks sensing)	IGF-1R, MET	lower levels in IGF-1R antisense-expressing cells [85], A-T cells display low levels of IGF-1R [86], MET-dependent ATM activation upon IR [98], increase in ATM autophosphorylation upon METi [99]
RAD51 (dsDNA breaks repair by homologous recombination)	IGF-1R, MET	intracellular trafficking controlled by IGF-1R [88], decrease in phosphorylation and nuclear translocation upon METi [100], interaction with BRCA2 hindered upon METi [101], reduced levels upon METi combined with IR [102]
BRCA2 (dsDNA breaks repair by homologous recombination)	MET	interaction with RAD51 hindered by METi [101]
γH2AX (dsDNA damage breaks sensing)	IGF-1R, MET	sustained γH2AX expression upon IGF-1R [89] and MET [99] inhibition, sustained γH2AX levels upon METi in combination with IR [99]

### 2.4. Activation of HGF-MET Axis Protects Cells from DDAs-Related Cytotoxicity

**Figure 1:**
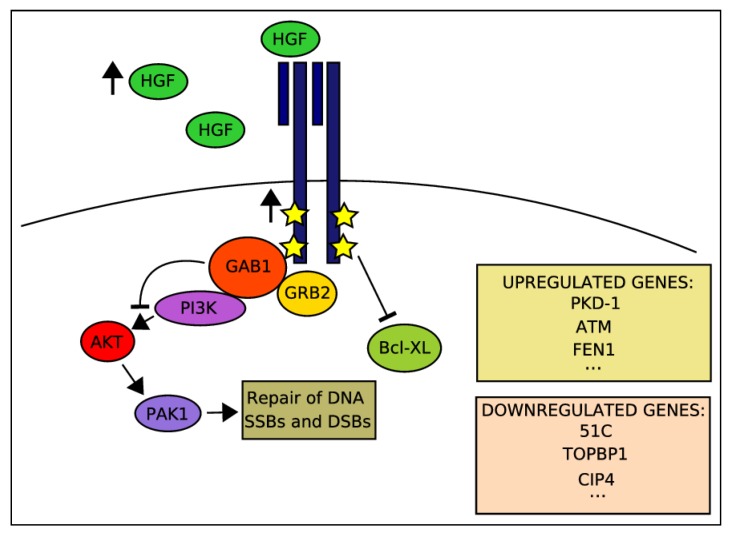
MET-downstream signaling activated by HGF stimulation prior to DNA damage and involved in MET-driven protection of cancer cell from DDAs.

The initial indications for a potential involvement of signaling by the MET RTK in cellular responses to DNA damage (Figure 1) emerged in the late 1990s with several publications by the group of Elliot Rosen. In 1998, Fan *et al*. reported an HGF-mediated tumor cells survival, which was correlated with decreased expression of the pro-apoptotic protein, Bcl-X_L_, following exposure to various DDAs, including IR and the topoisomerase IIα inhibitor adriamycin (ADR) [103]. In a subsequent study, the same group suggested that pre-treatment of human cancer cell lines with HGF enhanced in a PI3K-dependent manner their DNA repair activity towards both single and double strand DNA lesions [104]. Aiming at more mechanistic insights and by using the MDCK cell line as an experimental model, Fan *et al*. interestingly reported that the MET principal downstream signaling adaptor GAB1 is actually a negative regulator of HGF/MET-mediated DNA repair [105]. This inhibitory function did not require the GAB1 pleckstrin homology or SHP2 phosphatase-binding domain essential for MET/GAB1-mediated epithelial morphogenesis [106] but did require its PI3K-binding domain. In order to reveal alterations in gene expression that might contribute to HGF/MET-mediated cell protection and responses to DDAs, the same authors have used a cDNA microarray and a semi-quantitative RT-PCR approach, using the MDA-MB-453 human breast cancer cells that were incubated with HGF prior to ADR exposure [107]. Among various changes in gene expression patterns following HGF treatment, this study identified an induction of expression of the polycystic kidney disease 1 (PKD1) gene, which encodes a pro-survival constituent of cadherin-catenin complexes, which is downregulated upon ADR treatment. On the other hand, MET activation prior to ADR administration resulted in downregulation of such genes as 51C, an inositol polyphosphate 5-phosphatase, TOPBP1, which is a topoisomerase IIB-binding protein and the Rho-like small GTPase protein CIP4 [107]. A more detailed molecular significance of these changes in the context of a potential MET-DDR crosstalk has yet to be further investigated. In the studies that have been quoted so far, exogenous application of HGF has been shown to promote cellular survival towards DDAs. In a 2007 study, Sheng-Hua *et al*. showed a dose-dependent increase in HGF levels in the media of several glioma cells after a single dose radiation [108]. The authors identified that both baseline and radiation-enhanced HGF levels were about 10-fold higher in the BT325 cell line compared with other cell lines in the study. Interestingly, by employing a cell proliferation assay, the BT325 was more radioresistant than cell lines, which displayed a more moderate increase in IR-dependent HGF levels. As postoperative radiotherapy is a standard treatment for patients with malignant glioma and as these tumors display radioresistence and often relapse, even after a high dose of radiation, the authors suggested these *in vitro* observations as a potential relevant mechanism for HGF-mediated radioresistance of gliomas in clinical setups. In a similar work using pancreatic cell lines, Qian *et al*. monitored changes in MET levels and biologic activity following irradiation [109]. The authors reported an IR-related increased MET expression starting 3 hours post irradiation and reaching its peak 24 h later. Upon adding HGF, a MET-dependent MAPK activity associated with cellular scattering and invasion has been shown, however, without increase in cell proliferation [109].

### 2.5. Clinical Observations Correlating MET Expression with Responses to RT

While a large body of evidence supports tumor-associated MET signaling with cancer pathogenesis and progression [110,111], only few studies provided so far clues for a potential correlation between MET aberrant activity and responses to RT in clinical setups. In that respect, the 2001 report by Aebersold *et al*. was probably the first to suggest that MET expression is an independent and significant predicting factor of impaired local free-survival in a cohort of 97 patients with squamous cell carcinoma of the oropharynx who underwent definitive RT [112]. By employing an immunohistochemical analysis of various prognostic biomolecular markers in nasopharyngeal carcinoma treated by radiotherapy, Kim *et al*. identified high MET expression levels as a statistically significant negative prognostic factor on survival [113]. MET deregulated expression has been suggested to serve as a biomarker for tumor recurrence. Thus, overexpression of MET in hepatocellular carcinoma tissue or sustained high level of HGF in serum after hepatectomy were reported to relate with early tumor recurrence and metastasis [114]. Similarly, Raghav *et al*. reported in 2012 that high levels of phosphorylated MET are associated with increased risk of recurrence in patients with breast cancers [115]. Analogous observation regarding MET expression as a risk for tumor recurrence have been reported also for patients with carcinoma of the tongue [116].

In a 2000 manuscript, Di Renzo *et al*. reported a correlation between MET somatic mutations and lymph nodes metastasis in patients with head and neck squamous cell carcinomas (HNSCCs) [117]. As MET mutated variants exhibit also ligand-independent receptor enzymatic activity and since HGF was shown to protect tumor cells from DDAs [104], Aebersold *et al*. screened samples from HNSCC patients who were treated by radical RT for a correlation between presence of the MET Y1253D variant and responses to RT [118]. MET Y1253D has been identified in 15 out of a cohort of 138 patients. Both univariate and multivariate survival analysis revealed MET Y1253D to be significantly associated with impaired local tumor control. These findings provided evidence that the MET somatic mutation Y1253D is present in a notable subset of HNSCC patients and pointed out that this mutation may interfere with radioresponsiveness of these tumors, supporting further the notion of aberrant MET signaling as a target for radiosensitization.

### 2.6. Potentiation of IR-Induced Cytotoxicity by Targeting MET Signaling

MET targeting in cancer therapy has emerged as a major concept over the last decade with approximately 20 different selective as well as non-specific MET tyrosine kinase inhibitors (TKIs) and monoclonal antibodies that have been reported so far [119,120] out of which about 10 are currently in different phases of clinical trials [111]. Here, we will focus on the approaches that have been combined along with IR and that were discussed in the context of radiosensitization.

The Laterra group was the first to show in 2005 that targeting the MET pathway considerably potentiates tumor response towards IR [121]. By using anti-MET or anti-HGF ribozymes in a U87 glioblastoma xenograft model, the authors reported 0% response to irradiation alone and 20% response in animals that received intracranial delivery of the corresponding ribozymes. Animals that got a combined treatment exhibited an impressive 80% rate response. Moreover, combining the therapies led to tumor regression with a 40% tumor cure rate. Later, Chu *et al*. demonstrated both *in vitro* and *in vivo* and by using the U251 glioblastoma cell line a potentiation of IR-induced cytotoxicity by MET antisense oligonucleotides [122]. With respect to targeting MET pharmacologically, Welsh *et al*. found that the MP470 small molecule inhibitor considerably enhanced radiation-induced cell killing in SF767 cells [102]. Similarly, reduced clonogenic survival of gastric carcinoma cells GTL-16 has been reported by our group, using the indoline-2-one core anti-MET compound PHA665752 [99]. Other studies providing comparable data have used the MET small molecule inhibitors SU11274, MK-8003 and AMG548 [123,124,125], as well as the MET monoclonal antibody rilotumumab/AMG102 [126].

### 2.7. A Potential Molecular Crosstalk between MET and the DDR

**Figure 2:**
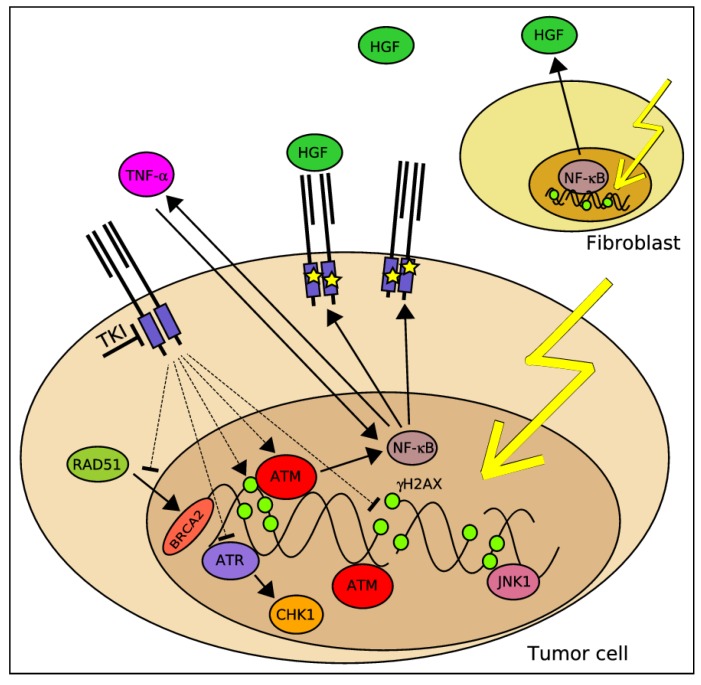
DDR sensors, mediators and effectors that have been shown to be modulated by MET signaling and/or MET inhibition alone or in combination with DDAs (*full lines—*direct links, *broken lines—*links that have not been proven direct).

Probably the most compelling data regarding how MET signaling may affect tumor resistance to DDAs, and in particular to IR, is related to the impact of MET signaling on the DDR apparatus, and in particular pathways involved in DNA repair (Table 1, Figure 2). In a manuscript published in 2008 [100], Ganapathipillai *et al*. suggested a signaling axis between mutated MET variants, the non-receptor tyrosine kinase ABL and the RAD51 recombinase, a critical HR DSBs repair effector [127]. The study showed that inhibition of MET tyrosine kinase activity by SU11274 was accompanied by reduction of phosphorylation of RAD51 on tyrosine 315, a residue whose phosphorylation has been associated with RAD51 nuclear translocation upon DNA damage [128,129]. Indeed, MET inhibition was further associated with impairment of nuclear translocation of a RAD51-GFP chimeric protein following irradiation. These events had also consequences on DNA repair as demonstrated by the ability of SU11274 to delay decline in γH2AX levels, indicating inhibition of DSBs repair [100]. In a follow-up paper, Medová *et al*. used a DR-GFP assay to demonstrate that MET targeting by PHA665752 or by specific siRNAs inhibited the ability of the corresponding cells to properly execute DSB repair via HR [101]. Mechanistically, treatment by the same drug has been found to disrupt the formation between RAD51 and BRCA2, an event, which is critical for HR to take place [101]. These data are very much similar with the aforementioned study by Li *et al*. who reported HR inhibition via EGFR inhibition by erlotinib [68]. Another indication for the negative impact of MET inhibition on DSBs repair has been provided by Welsh *et al*. who reported increased γH2AX foci with reduced RAD51 levels upon combination of IR with the small molecule inhibitor MP470 [102]. In an attempt to investigate in further details the interaction between MET and the DDR, Medová *et al*. described in a 2010 study a synergistic mode of interaction between PHA665752 and IR [99]. Importantly, it has also been found, as a potential molecular mechanism underlying this synergism, that MET inhibition alone was sufficient to increase γH2AX levels in GTL-16 gastric cancer cells, strongly indicating that MET signaling may be involved in repair of DNA damage of endogenous replicative stress origin [99]. If further confirmed, such a finding may at least partially account for the synergism between MET targeting and IR, as inhibition of the DNA repair driven via MET may compromise the ability of cells to repair the damage inflicted via irradiation.

In two *Nature* articles published in 2009, Xiao *et al*. and Cook *et al*. reported tyrosine 142 as a novel regulatory site of H2AX whose phosphorylation and subsequent dephosphorylation are executed by the WIHC complex and the EYA1/3 phosphatases, respectively [130,131]. H2AX tyrosine phosphorylation was suggested to function as a novel regulatory mechanism, which affects histone associations with either pro-apoptotic or repair factors. Overall, lasting tyrosine phosphorylation was shown to correlate with H2AX recruitment of pro-apoptotic effectors such as the JNK1 kinase, eventually leading to apoptosis. Interestingly, when MET activity was targeted in GTL-16 by PHA665752, alone or in combination with irradiation, a massive increase of H2AX phosphorylation on tyrosine 142 with the subsequent interaction with the pro-apoptotic kinase JNK1 has been observed, providing therefore an additional mechanistic aspect of how MET inhibition contributes to a compromised cellular response towards IR.

More supportive findings on the potential role of MET for cellular responses to DDAs has been recently provided by De Bacco *et al*. who demonstrated that IR affects the expression and activity of MET [98]. After irradiation, MET expression in breast cancer cell lines was increased up to fivefold, and this overexpression increased ligand-independent MET phosphorylation and signal transduction, which protects cells from apoptosis [98]. Cells that survived irradiation also became increasingly invasive, an effect that was driven through activation of ATM and nuclear factor κB (NF-κB) signaling pathways. 

Finally and in addition to that effects that MET inhibition has on DNA repair, a 2010 study by Medová *et al*. investigated potential impact of PHA665752 on the checkpoint control arm of the DDR [99]. In that context, this drug showed a MET-dependent inhibition of ATR, CHK1, and CDC25B with consequent abrogation of an associated DNA damage–induced S phase arrest. This may indicate that MET inhibition compromises a critical damage dependent checkpoint that may enable premature exit of DNA-damaged cells from cell cycle arrest before repair is completed.

### 2.8. The Relevance of MET Signaling to Tumor Resistance towards RT under Hypoxic Conditions

Poor oxygenation is a common feature of solid tumors. On one hand, deregulated growth overrides the ability of the vasculature to adapt to the increased oxygen demand [132,133]; on the other, tumor blood vessels are functionally impaired compared to normal tissues due to structural and biological abnormalities such as leakiness and lack of pericytes [134]. As a result, neoplastic lesions often contain areas subjected to acute or chronic hypoxia regardless of blood vessel proximity [135].

Oxygen insufficiency limits the growth of the tumor, because cancer cells, as other cells in the organism, utilize oxygen to generate energy and as a substrate for a number of fundamental biochemical processes including synthesis of macromolecules. On the contrary, however, and somehow in contrast to expectations, overwhelming experimental and clinical evidence suggests that hypoxic tumors are more aggressive than well-oxygenized lesions and that their prognosis is less favorable [34,136,137,138,139,140]. This is currently attributed to the fact that hypoxia induces a number of cellular adaptations that may turn advantageous during tumor progression, including a switch to anaerobic metabolism [141], increased genetic instability [142], promotion of angiogenesis [143], activation of invasive growth [13] and preservation of stem cells features [144].

Apart of its impact on tumor growth and progression, hypoxia also represents a major obstacle for radiotherapy as hypoxic cells are approximately three-fold more resistant than well-oxygenated cells [140]. Furthermore, hypoxia has similar resistance effects on the response of tumors to anti-cancer drugs that elicit their pharmacological effect on oxygen-generated free radical species [145].

The biological effect of radiation depends on the degree of oxygenation. This oxygen effect is due to the interaction between oxygen and the free radicals produced when radiation is absorbed in tissues. These highly reactive, short-lived free radicals produce DNA DSBs, leading eventually to cell death. Oxygen amplifies the damage produced by radiation by increasing the lifetime of free radicals. Due to their short life-span, oxygen needs to be present at the time of irradiation to be effective [146,147]. In that respect, several studies showed poor locoregional control and survival in patients with hypoxic tumors treated with radiation [139,148,149,150,151,152].

Since the discovery of MET and its ligand HGF, much research interest has focused on their roles in cancer [153]. Importantly, MET transcriptional activation and deregulated signaling were also shown to have a role in the biology of tumor-associated hypoxia. In 2003, Pennacchietti *et al*. discovered a molecular mechanism linking hypoxia to increased transcription of the *MET* gene whose promoter contains several HIF1α-responsive elements [13]. Concomitant to increased expression, upregulation of the MET kinase and its signaling activity have been linked to a deregulated MET-dependent invasive growth program, a finding that could correlate with an increased metastatic potential of hypoxic tumors. In further line with these observations, Hara and colleagues reported in 2006 that HIF-1α siRNA decreased the synergistic activities of hypoxia and HGF on cellular invasion [154]. 

With respect to the relevance of MET aberrant expression to tumor-related hypoxia in both preclinical and clinical setups, in human cervical cancer xenografts as well as in human breast tumor samples, MET overexpression positively correlated with presence of hypoxic regions [13,155]. Likewise, elevated MET and HIF-1α mRNA expression was observed at the invading front in well-differentiated thyroid papillary carcinoma [156]. HIF-1α expression was also found to significantly correlate with stromal HGF expression and MET expression in human pancreatic adenocarcinoma [157]. Furthermore, MET and HIF-1α expression were reported as independent predictors for distant metastasis and correlated with a poor 10-year disease-free survival rate in breast cancer patients [155]. These studies suggest that hypoxia may enhance signaling of the HGF-MET axis and thereby promote cancer cell metastasis through upregulation of MET expression *in vivo*. Hypoxia has also been shown to increase HGF-MET signaling, scattering, and invasive activity of cancer cells *in vitro*. Hypoxic upregulation of *MET* promoter activity, MET expression and phosphorylation have also been observed in cell lines derived from normal tissues as well as tumors [13,157], while HGF expression and secretion were reported to be increased in hypoxic fibroblasts [157].

Taken together, although tumor hypoxia is known as a critical negative determinant of radiation therapy outcome in several human cancers, the full picture of molecular pathways that contribute to tumor hypoxia-mediated radioresistance remains largely elusive and a better insight into MET signaling under hypoxia with particular emphasis on the receptor crosstalk with DDR pathways and functioning as well as evaluation of the potential of MET inhibitors to increase tumor cells response to therapeutically-relevant irradiation doses under hypoxic conditions needs to be investigated in further details.

## 3. Conclusions

The MET system whose aberrant activity is found in numerous types of human cancers is considered as a prime target in clinical oncology. In addition to obvious physiological traits that are associated with oncogenic activity of a growth factor receptor such as uncontrolled proliferation, tumor cell survival, local aberrant invasion with eventual systemic dissemination, accumulating data from various studies support the existence of a signaling between MET and the DDR, resembling in that respect other RTKs and in particular, the EGFR.

Apart of extending existing paradigms over the biology of MET as a “classical” growth factor receptor that may also be involved in maintenance of genomic stability, such observations are also critically important with respect to clinical circumstances. Thus, MET may not only serve as a major determinant of cancer progression in terms of local growth and metastasis, but it may also be an important signaling regulator of responses to DNA damage that can “assist” tumor cells evading RT-induced cytotoxicity. This assumption, that has still to be further confirmed, provides a strong biological rationale for targeting MET in order to obtain more optimal responses to RT and potentially other DDAs of MET expressing tumors. Still, many questions remain at large open and much more studies are required in order to identify the details of the particular pathways through which MET is signaling and wired with the DDR.
